# Associations between dietary risk factors and ischemic stroke: a comparison of regression methods using data from the Multi-Ethnic Study of Atherosclerosis

**DOI:** 10.4178/epih.e2018021

**Published:** 2018-05-21

**Authors:** Seyed Saeed Hashemi Nazari, Yaser Mokhayeri, Mohammad Ali Mansournia, Soheila Khodakarim, Hamid Soori

**Affiliations:** 1 Department of Epidemiology, School of Public Health, Shahid Beheshti University of Medical Sciences, Tehran, Iran; 2 Safety Promotion and Injury Prevention Research Center, Shahid Beheshti University of Medical Sciences, Tehran, Iran; 3 Department of Epidemiology and Biostatistics, School of Public Health, Tehran University of Medical Sciences, Tehran, Iran; 4 Department of Epidemiology, School of Paramedical Science, School of Public Health, Shahid Beheshti University of Medical Sciences, Tehran, Iran

**Keywords:** Stroke, Risk factors, Diet, Multi-Ethnic Study of Atherosclerosis

## Abstract

**OBJECTIVES:**

We analyzed dietary patterns using reduced rank regression (RRR), and assessed how well the scores extracted by RRR predicted stroke in comparison to the scores produced by partial least squares and principal component regression models.

**METHODS:**

Dietary data at baseline were used to extract dietary patterns using the 3 methods, along with 4 response variables: body mass index, fibrinogen, interleukin-6, and low-density lipoprotein cholesterol. The analyses were based on 5,468 males and females aged 45-84 years who had no clinical cardiovascular disease, using data from the Multi-Ethnic Study of Atherosclerosis.

**RESULTS:**

The primary factor derived by RRR was positively associated with stroke incidence in both models. The first model was adjusted for sex and race and the second model was adjusted for the variables in model 1 as well as smoking, physical activity, family and sibling history of stroke, the use of any lipid-lowering medication, the use of any anti-hypertensive medication, hypertension, and history of myocardial infarction (model 1: hazard ratio [HR], 7.49; 95% confidence interval [CI], 1.66 to 33.69; p for trend=0.01; model 2: HR, 6.83; 95% CI, 1.51 to 30.87 for quintile 5 compared with the reference category; p for trend=0.02).

**CONCLUSIONS:**

Based primarily on RRR, we identified that a dietary pattern high in fats and oils, poultry, non-diet soda, processed meat, tomatoes, legumes, chicken, tuna and egg salad, and fried potatoes and low in dark-yellow and cruciferous vegetables may increase the incidence of ischemic stroke.

## INTRODUCTION

Although from 2000 to 2010, age-adjusted mortality due to stroke declined by 36% [1], as of 1910 it remained among the 5 leading causes of death in the US [2]; furthermore, stroke has been classified as one of the main reasons for reduction in active life expectancy [3]. According to numerous studies, diet has a considerable effect on stroke incidence [4,5]. Other studies have reported a negative association between certain dietary factors and stroke incidence [6].

Some researchers have found that an enhanced inflammatory response may lead to the development of stroke [7]. The concentration of inflammatory markers in stroke patients usually is higher than in healthy subjects. Generally, inflammatory markers such as interleukin (IL)-6 result in arterial stiffness. Moreover, arterial stiffness is a known predictor of cardiovascular disease (CVD) [8,9]. Additionally, fibrinogen, as a clotting factor, could bring about platelet aggregation, resulting in thrombus formation [10]. Low-density lipoprotein (LDL) cholesterol [11] and body mass index (BMI) [12] have proven useful for predicting stroke incidence. Elevated LDL levels lead to atherosclerosis through a mechanism involving endothelial dysfunction, inflammation, and a procoagulant vascular surface [13]. Moreover, visceral fat accumulation may cause increased lipid synthesis, resulting in hyperlipidemia and atherosclerosis [14].

The associations between diet and health outcomes are not a novel topic of research, and many methods have been developed to study this issue [15,16]. Food materials are consumed in combination, and the usual scoring approaches do not consider the correlational structure of food items in deriving scores for nutritional quality. Hence, these methods fail to derive correct dietary patterns for predicting outcomes. In contrast, exploratory methods such as ordinary principal component analysis ignore nutritional information selected aspects of diet and simply derive a score on the basis of correlations and linear associations between dietary items [17].

Recently, some hybrid methods have been established, such as reduced rank regression (RRR) and maximum redundancy analysis. These hybrid methods consider not only nutritional information selected aspects of diet, but also take into account the correlational structure of food and nutrient intake. By including some intermediate factors, also known as response variables (factors that have a reasonably strong association with the final outcome, such as risk factors, nutrient intake, biomarkers, or even ratios of nutrients and biomarkers), RRR is used to derive dietary intake scores indicative of factors contributing to the development of the final outcome [17].

Some studies have investigated the association between dietary patterns and stroke, but none have applied RRR [4,18]. In this study, we extracted dietary patterns using RRR, as a hybrid method (with BMI, fibrinogen, IL-6, and LDL as response [intermediate] variables), and aimed to assess how well the scores extracted by RRR predicted stroke in comparison to those produced by partial least squares (PLS) and principal component regression (PCR) models.

## MATERIALS AND METHODS

### Study population

In July 2000, the Multi-Ethnic Study of Atherosclerosis (MESA), a population-based study, was designed to identify characteristics related to the progression of subclinical to clinical CVD [19]. In brief, the MESA recruited 6,814 males and females aged 45-84 years who had no clinical CVD. The study subjects were recruited in a way that ensured ethnic diversity, including Hispanic, Chinese (Asian), Black, and Caucasian (White) subjects from 6 field centers including Forsyth County, NC; Northern Manhattan and the Bronx, NY; Baltimore City and Baltimore County, MD; St. Paul, MN; Chicago and the village of Maywood, IL; and Los Angeles County, CA. Written informed consent was provided by all participants.

**Figure 1.**
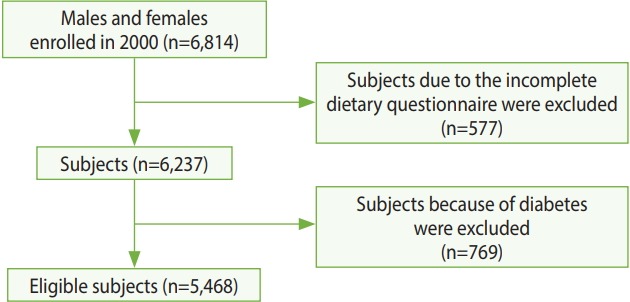
Flowchart for selection of study participants from the Multi-Ethnic Study of Atherosclerosis.

In this cohort study, 577 subjects were excluded due to having an incomplete dietary questionnaire and 769 subjects were excluded because of diabetes (according to the American Diabetes Association 2003 criteria). Finally, 5,468 males and females were analyzed. Figure 1 shows the flowchart for the selection of study participants. We used the first 3 phases of the MESA because those phases were available to us. The MESA started in 2000 and is an ongoing population-based cohort study.

### Follow-up and ascertainment of stroke (outcome event)

The follow-up period was defined as extending from July 15, 2000 to July 15, 2005 (five years).

According to the MESA protocol, the outcome—stroke—was classified as present or absent and consisted of rapid onset of a documented focal neurologic deficit lasting 24 hours or until death, or if the deficit lasted < 24 hours, there was a clinically relevant lesion on brain imaging. Patients with focal neurologic deficits secondary to brain trauma, tumor, infection, or other non-vascular causes were excluded [20].

In our research, only ischemic stroke was included in the analysis, and hemorrhagic and transient ischemic attacks were excluded.

### Low-density lipoprotein, interleukin-6, fibrinogen, and body mass index

We used these 4 response variables to derive dietary patterns using RRR. Data for LDL and 2 inflammatory markers—IL-6 and fibrinogen—were measured in blood samples in the first phase. Previous research has revealed an association between LDL levels and stroke [21]. In addition, the inflammatory markers IL-6 [22] and fibrinogen [23] could be triggers for developing stroke in later years. We also included BMI, which has shown a positive effect on stroke risk [24-26]. BMI was measured by the ratio of weight (kg) to height squared (m^2^ ).

### Other relevant variables

In order to eliminate the effect of confounders, we included the following demographic and lifestyle covariates in the statistical models: age, sex, race, smoking, physical activity (intentional), parental history of stroke, sibling history of stroke, the use of any lipid-lowering medication, the use of any anti-hypertensive medication, hypertension, and history of myocardial infarction.

### Dietary assessment

At baseline, the MESA used a 120-item food-frequency questionnaire (FFQ), which was developed and validated by Block et al. [27] to assess the average frequency of intake (9 frequency options), ranging from “rare or never” to “2+ per day” for food and “6+ per day” for beverages, and serving size (small, medium, or large) during last 12 months. The 120 items were categorized into 47 food groups.

### Statistical methods

In this study, we derived dietary patterns from 47 food groups using 3 methods: RRR, PCR, and PLS. For this purpose, we used 2 sets of variables: predictor variables, defined as the 47 food groups, and the response variables, which were defined as BMI, LDL, IL-6, and fibrinogen. Due to the non-normal distribution of the response variables, natural logarithm values were applied [28]. All 3 methods seek to extract some predictors (components) using successive linear combinations, but they do not use the same assumptions. Briefly, RRR seeks to extract patterns in the predictors (in this case, dietary patterns) that maximize the explained variation in the response variables. Inversely, PCR tries to derive factors that explain as much variation as possible in the predictors (47 food groups), and PLS strikes a balance of the 2 aims, seeking for factors that explain both response and predictor variation [17]. The optimal number of extracted factors is determined using the cross-validation method (split method). The optimal number of extracted factors from RRR, PCR, and PLS was the same because all 3 methods were included in the PLS procedure. All statistical analyses were performed using SAS version 9.4 (SAS Institute Inc., Cary, NC, USA).

We also used a Cox proportional hazard (PH) model to investigate the relationship between stroke and derived dietary patterns (score variables). In the Cox model, age was considered as the origin of time and follow-up times were considered from the date of entry into the study. In this way, left truncation was accounted for in the analyses [29]. To test the PH assumption, we used both the goodness-of-fit test and the interaction of variables with time. None of the predictor variables violated the PH assumption. The hazard ratios (HR) for stroke incidence were calculated for quintiles 2, 3, 4, and 5 of the score variables, considering quintile 1 as the reference category. We ran 2 models for analyses. The first model was adjusted for sex (male or female) and race (White, Black, Chinese or Hispanic) and the second model was adjusted for the variables in model 1 as well as smoking (never, former, or current), physical activity (total intentional exercise, metabolic equivalent-hr/wk), family history of stroke (parental, none, yes, or do not know), sibling history of stroke (none, yes, not applicable, or do not know), the use of any lipid-lowering medication (yes or no), the use of any anti-hypertensive medication (yes or no), hypertension (yes or no), and history of myocardial infarction. The interactions between the dietary pattern score and race, sex, physical activity, and smoking were assessed. Furthermore, the p-values for trends were estimated by treating the dietary pattern scores as a linear term. Cox analyses were carried out with Stata version 12 (StataCorp., College Station, TX, USA).

Finally, we singled out the top 10 food groups that contributed the maximum variation—whether positive or negative—in the derived dietary pattern scores. For this purpose, the unadjusted Pearson correlation coefficient between each food group and each dietary pattern score was calculated. Then, the standardized β regression coefficient between each food group and each dietary pattern was estimated. Eventually, by multiplying these 2 summary measures, the explained proportion of score variation was obtained for each of the 10 food groups [17,28].

### Ethical approval

For this study, we did not require any ethical approval because the data were acquired from the National Heart, Lung, and Blood Institute (NHLBI)—Research Materials Distribution Agreement V02 1d20120806.

## RESULTS

A total of 5,468 participants (46.4% males) were studied. Their mean age was 61.8± 10.3 years. After 26,145 person-years of follow-up, 47 new cases of ischemic stroke occurred. The incidence rate of stroke was 17.97 per 10,000 person-years.

Since the first factor is usually considered to be the most noteworthy, to summarize the results, we only presented the outputs for the primary factor derived by RRR (RRR 1), PCR (PCR 1), and PLS (PLS 1). The participants’ characteristics across the quintiles are compared in Table 1. According to all 3 methods, the higher the dietary pattern score, the more likely the participant was to be a smoker, to be Black, to have a higher BMI, and to have a higher concentration of IL-6. Age was negatively associated with the dietary pattern score in all 3 methods. Stroke incidence was positively associated with the dietary pattern score for RRR 1. Despite these similarities, there were some discrepancies among the methods. For instance, sex (males) in PCR 1 and PLS 1 was positively associated with the dietary score, while no marked trend was observed in RRR 1. Moreover, subjects with a higher dietary pattern score derived using PCR 1 and PLS 1 showed less physical activity (intentional exercise), but no clear trend was found in RRR 1.

Regarding the contribution of each statistical method to explaining variation in food groups (predictors) and responses, the maximum and minimum variation in food groups were explained by PCR (26.95%) and RRR (13.97%), respectively. In contrast, RRR accounted for the highest variation in the response variables (5.33%), in contrast to PCR, which accounted for 2.37% of the variation. The variation explained by PLS for both food groups and responses was between PCR and RRR; in general, the results for PLS reflected a balance between the 2 other methods (Table 2).

The results of the Cox PH analyses are illustrated in Table 3. A significant association between dietary pattern and stroke incidence was only found for RRR 1 in both models (adjusted for sex and race in model 1 and adjusted for sex, race, smoking, physical activity [total intentional exercise], family history of stroke [parent], family history of stroke [sibling], any lipid-lowering medication, any anti-hypertensive medication, hypertension, and history of myocardial infarction in model 2). In RRR 1 in model 1, the hazard of stroke among subjects in the fifth quintile was more than 7 times higher than that of the subjects in the first quintile (hazard ratio [HR], 7.49; 95% confidence interval [CI], 1.66 to 33.69). In RRR 1 in model 2, the hazard of stroke among subjects in the fifth quintile was also approximately 7-fold higher than that of the subjects in the first quintile (HR, 6.83; 95% CI, 1.51 to 30.87). In contrast, neither PCR 1 nor PLS 1 was significantly associated with stroke incidence. None of the interaction terms between dietary pattern scores and race, sex, physical activity, and smoking were statistically significant.

Tables 4 presents the 10 food groups that contributed maximally to the first dietary pattern scores obtained by RRR, PCR, and PLS, respectively. The first 10 food groups contributed to 79.08% of variation in the first factor extracted using the RRR method. Additionally, 55.41% of variation in PCR 1 (the first factor extracted by PCR), and 59.64% of variation in PLS 1 (the first factor extracted by PLS) were explained by the first 10 food groups. In all 3 methods, fats and oils, poultry, processed meat, tomatoes, and fried potatoes were positively correlated with the dietary pattern score. Cruciferous vegetables and dark-yellow vegetables were negatively correlated factors in the first 10 food groups according to RRR 1 and PLS 1. No protective factors were found among the 10 food groups using PCR 1.

## DISCUSSION

In this study, the authors aimed to identify food groups related to stroke incidence using 3 methods (RRR, PCR, and PLS), and to assess how well the score extracted by RRR predicted stroke in comparison to those produced by PCR and PLS. We observed that 79.08% of variance in RRR 1 could be explained by the first 10 food groups. Both RRR 1 and PLS 1 determined fats and oils to be the most important contributors that accounted for the most variation in dietary patterns, while PCR 1 showed red meat to be the main food group. In both models, only RRR 1 yielded positive, significant associations with stroke incidence.

Our findings regarding saturated fatty acids agree with those of a study by Yamagishi et al. [30] that demonstrated a positive effect of saturated fatty acids on stroke incidence over 22 years of follow-up. Micha & Mozaffarian [31] reviewed a range of randomized controlled trials and prospective cohort studies. They reported that consumption of polyunsaturated fats, in preference to saturated fatty acids, could reduce the risk of coronary heart disease, but they did not find a clear effect on stroke.

Our results regarding red meat are also consistent with those of other studies. Yang et al. [32], based on a systematic review and meta-analysis, observed a dose-response association between red meat consumption (especially processed red meat) and risk of total stroke (relative risk, 1.14; 95% CI, 1.05 to 1.24). Although red meat is traditionally considered as a rich source of iron, protein, zinc, and other nutrients, it might contain some unhealthy compounds. High consumption of red meat could result in imbalanced serum lipid profiles because it contains saturated fatty acids and cholesterol [33]. Moreover, heme iron plays a role in the formation of N-nitrosation compounds [34]. An epidemiological study revealed a relationship between these compounds and CVD [35].

Even though using all 3 methods, poultry was classified in the first 10 food groups that were positively associated with the first dietary pattern, studies have not indicated a significant association between poultry and stroke [36]; in fact, Bernstein et al. [37] found a protective effect of poultry against stroke.

According to our study, both RRR 1 and PLS 1 found that darkyellow vegetables and cruciferous vegetables were among the 10 food groups that explained the most variation in the first dietary pattern score. They had a high negative correlation with the first dietary pattern, meaning that they exerted a protective effect against stroke incidence. Borgi et al. [38], reviewed 3 prospective cohort studies of US males and females to explore the association of fruits and vegetables with hypertension, which is a well-known risk factor for stroke. Despite the non-significant effect of total vegetables, their analysis of individual vegetables revealed that broccoli and carrots (classified as cruciferous and dark-yellow vegetables, respectively) were associated with a lower risk of hypertension [38]. Moreover, a meta-analysis of prospective cohort studies demonstrated that vegetable consumption could reduce the risk of stroke [39]. This reduction might be explained by biological mechanisms such as lowering blood pressure, improving microvascular function [40], and a modifying effect on BMI and total cholesterol [41].

In general, RRR has emerged as a valuable and powerful tool for deriving dietary patterns in nutritional epidemiology. The RRR method uses both prior knowledge and study data. Using disease-specific response variables, RRR can be utilized for biological and etiological investigations. Therefore, using RRR, the pathways between diet and disease can be evaluated. Moreover, RRR is more flexible than PCR because it works with 2 sets of variables. Incorporation of prior knowledge is generally considered the most interesting advantage of the RRR method compared with PCR. The RRR combines the strength of PCR—an assessment of the correlational structure of food groups—with the advantage of using response variables to predict the final outcome [17].

A few shortcomings of this study should be acknowledged. First, dietary data were collected using a self-reported instrument (the FFQ) that is subject to measurement error. Second, assessing diet during a year through only a single FFQ cannot be reliable. Participants’ diet might have changed within a year, potentially affecting our results regarding stroke incidence. Third, the choice of response variables was somewhat arbitrary, and a different choice might yield different results. Fourth, estimate inflation might have occurred due to the small number of outcome events (47 new ischemic stroke cases), leading to sparse data bias [42].

In conclusion, RRR extracted a more predictive dietary pattern linked to the outcome variables than did PCR and PLS. We found that a dietary pattern high in fats and oil, poultry, non-diet soda, processed meat, tomatoes, legumes, chicken, tuna and egg salad, and fried potatoes and low in dark-yellow and cruciferous vegetables may increase the incidence of stroke.

## Figures and Tables

**Table 1.** Descriptive characteristics according to the primary dietary pattern generated using RRR, PCR, and PLS in 5,468 males and females from MESA^1^

	Dietary pattern											
	RRR 1				PCR 1				PLS 1			
	Q1 (n=1,094)	Q3 (n=1,093)	Q5 (n=1,093)	p for trend	Q1 (n=1,094)	Q3 (n=1,093)	Q5 (n=1,093)	p for trend	Q1 (n=1,094)	Q3 (n=1,093)	Q5 (n=1,093)	p for trend
Male (%)	49.4	42.3	47.4	0.59	36.9	47.1	52.8	<0.001	39.1	44.4	55.8	<0.001
Age (yr)	62.3±10.3	62.5±10.4	60.1±9.9	<0.001	63.3±10.1	62.5±10.1	59.6±10.5	<0.001	62.9±10.2	62.5±10.4	59.2±10.1	<0.001
Smoker (%)	8.5	12.2	17.5	<0.001	9.6	12.1	18.1	<0.001	5.1	11.7	21.0	<0.001
Exercise (MET-hr/wk)^2^	27.3±36.9	27.5±39.7	22.9±36.6	0.004	25.5±40.2	26.3±37.3	25.9±36.5	0.87	28.6±40.3	26.2±37.4	23.7±35.5	<0.001
Srroke (%)												
Parent	21.9	29.4	30.0	0.001	28.7	29	27.9	0.98	24.1	27.4	29.9	0.007
Sibling	6.4	9.5	8.5	0.05	8.6	7.5	6.7	0.02	7.0	8.7	6.5	0.61
Myocardial infarction (%)	1.4	2.5	2.1	0.11	2.4	2.2	1.3	0.21	1.4	1.8	2.0	0.36
Lipid medication (%)	13.1	15.1	10.8	0.06	16.5	16.2	11.5	<0.001	14.6	15.6	10.2	0.003
HBP medication (%)^3^	28.1	35.9	33.1	0.006	37.6	31.8	31.2	0.001	30.2	34.9	30.9	0.43
Hypertension (%)	21.8	25.3	23.8	0.31	27.0	23.3	22.3	0.005	24	23.8	21.2	0.09
Ethnic diversity (%)												
White	29.8	50.2	37.1	0.03	31.3	49.8	42.4	<0.001	21.2	49.9	45.7	<0.001
Chinese	54.4	1.0	0.1	<0.001	22.3	11.1	5.1	<0.001	55.9	1.4	0.2	<0.001
Black	9.3	27.3	33.9	<0.001	25.4	21.0	28.7	0.05	14.9	25.9	29.1	<0.001
Hispanic	6.4	21.4	28.8	<0.001	20.8	17.9	23.7	0.35	7.8	22.6	25	<0.001
LDL (mg/dL)^4^	112 (110, 113)	114 (112, 116)	115 (113, 117)	0.005	112 (110, 114)	112 (110, 114)	114 (112, 116)	0.49	111 (109, 113)	112 (110, 114)	115 (113, 117)	0.004
BMI (kg/m^2^)^4^	24.8 (24.6, 25.0)	27.7 (27.4, 28.0)	29.7 (29.3, 30.0)	<0.001	26.3 (26.1, 26.6)	27.1 (26.8, 27.4)	28.7 (28.4, 29)	<0.001	24.8 (24.5, 25)	27.6 (27.3, 27.9)	29.2 (28.9, 29.5)	<0.001
Fibrinogen (mg/dL)^4^	322 (318, 326)	336 (332, 340)	345 (341, 349)	<0.001	337 (333, 341)	334 (330, 338)	334 (330, 338)	0.20	328 (324, 332)	336 (331, 340)	336 (332, 341)	0.01
IL-6 (pg/mL)^4^	0.95 (0.92, 0.99)	1.25 (1.20, 1.30)	1.36 (1.31, 1.41)	<0.001	1.14 (1, 1.18)	1.15 (1.11, 1.20)	1.24 (1.20, 1.29)	0.02	0.96 (0.92, 0.99)	1.22 (1.17, 1.27)	1.31 (1.26, 1.36)	<0.001
Stroke (%)	0.18	0.91	1.10	0.05	0.73	1.56	0.73	0.84	0.46	0.91	0.64	0.75

0.75RRR, reduced rank regression; PCR, principal component regression; PLS, partial least squares; MESA, Multi-Ethnic Study of Atherosclerosis; Q, quintile; MET, metabolic equivalents; HBP, high blood pressure; LDL, low-density lipoprotein; BMI, body mass index; IL, interleukin.

1 The test for trend was run by treating the score variable as a linear term.

2 Total intentional exercise (MET-hr/wk).

3 Any anti-hypertensive medication.

4 Geometric means due to log-transformed values.

**Table 2.** Explained variation in food groups and responses by 3 methods from MESA data

Factor	Explained variation in food groups (%)^1^			Explained variation in responses (%)^2^		
	PCR	PLS	RRR	PCR	PLS	RRR
1	11.98	9.55	5.56	0.70	2.61	3.75
2	6.96	7.32	3.44	1.46	0.88	0.88
3	4.22	4.42	2.26	0.06	0.60	0.41
4	3.79	2.56	2.71	0.15	0.54	0.29
Total	26.95	23.85	13.97	2.37	4.63	5.33

MESA, Multi-Ethnic Study of Atherosclerosis; PCR, principal component regression; PLS, partial least squares; RRR, reduced rank regression.

1 All food items were categorized into 47 food groups.

2 The selected responses are body mass index, interleukin-6, fibrinogen and low-density lipoprotein.

**Table 3.** Hazard ratios and 95% confidence intervals for stroke by 2 models^1^ and 3 methods according to quintiles of the first dietary pattern score in 5,468 males and females from MESA

	Quintile 1	Quintile 2	Quintile 3	Quintile 4	Quintile 5	p for trend^2^
RRR 1						
Model 1	1.00 (reference)	5.69 (1.27, 25.46)	4.84 (1.06, 22.16)	5.97 (1.32, 27.02)	7.49 (1.66, 33.69)	0.01
Model 2	1.00 (reference)	5.66 (1.26, 25.38)	4.87 (1.06, 22.34)	5.97 (1.31, 27.07)	6.83 (1.51, 30.87)	0.02
PCR 1						
Model 1	1.00 (reference)	1.07 (0.40, 2.86)	2.51 (1.07, 5.87)	1.03 (0.35, 3.01)	1.45 (0.54, 3.91)	0.45
Model 2	1.00 (reference)	1.16 (0.43, 3.11)	2.65 (1.13, 6.18)	1.10 (0.37, 3.23)	1.47 (0.54, 3.96)	0.44
PLS 1						
Model 1	1.00 (reference)	2.46 (0.87, 6.92)	2.13 (0.72, 6.25)	3.14 (1.10, 8.97)	2.07 (0.65, 6.59)	0.17
Model 2	1.00 (reference)	2.51 (0.88, 7.09)	2.18 (0.74, 6.45)	3.08 (1.07, 8.88)	2.02 (0.63, 6.64)	0.20

MESA, Multi-Ethnic Study of Atherosclerosis; RRR 1, the primary factor derived by, reduced rank regression; PCR 1, the primary factor derived by, principal component regression; PLS 1, the primary factor derived by, partial least squares.

1 Cox proportional hazard model adjusted for sex (male or female) and race (White, Black, Chinese or Hispanic) in model 1 and also adjusted for sex, race, smoking (never, former or current), physical activity (total intentional exercise) (MET-hr/wk), family history of stroke (parent) (no, yes, or don’t know), family history of stroke (sibling) (no, yes, not applicable, or don’t know), any lipid-lowering medication (yes or no), any anti-hypertensive medication (yes or no), hypertension (yes or no), and myocardial infarction (yes or no) in model 2.

2 p for trend was obtained by treating the score variable as a linear term.

**Table 4.** The 10 food groups most strongly associated with the first dietary pattern obtained by RRR, PCR and PLS in 5,468 males and females from the MESA

	Correlation coefficient^1^	Regression coefficient^2^	Contribution to total score variance (%)^3^
RRR factor 1			
Food groups with positive correlations			
Fats and oils	0.47	0.26	12.65
Poultry	0.34	0.27	9.66
Non-diet soda	0.43	0.22	9.52
Processed meat	0.43	0.20	8.78
Tomatoes	0.33	0.22	7.37
Legumes	0.30	0.20	6.29
Chicken, tuna, and egg salad	0.27	0.14	3.93
Fried potatoes	0.38	0.09	3.60
Food groups with negative correlations			
Dark-yellow vegetables	-0.42	-0.25	10.98
Cruciferous vegetables	-0.44	-0.14	6.30
All 10 food groups			79.08
PCR factor 1			
Food groups with positive correlations			
Red meat	0.68	0.12	8.44
High-fat cheeses and sauces	0.63	0.11	7.17
Poultry	0.60	0.10	6.46
White bread	0.60	0.10	6.40
Tomatoes	0.57	0.10	5.85
Fats and oils	0.56	0.09	5.57
Fried potatoes	0.48	0.08	4.17
Processed meat	0.48	0.08	4.13
Other vegetables	0.45	0.08	3.62
Potato and pasta salad	0.44	0.08	3.60
All 10 food groups			55.41
PLS factor 1			
Food groups with positive correlations			
Fats and oils	0.61	0.14	8.95
Processed meat	0.56	0.13	7.66
High-fat cheeses and sauces	0.62	0.12	7.57
Fried potatoes	0.56	0.12	7.30
Non-diet soda	0.42	0.13	5.73
Tomatoes	0.44	0.10	4.75
Desserts	0.46	0.09	4.58
Poultry	0.41	0.10	4.44
Food groups with negative correlations			
Cruciferous vegetables	-0.35	-0.14	5.03
Dark-yellow vegetables	-0.27	-0.13	3.63
All 10 food groups			59.64

RRR, reduced rank regression; PCR, principal component regression; PLS, partial least squares; MESA, Multi-Ethnic Study of Atherosclerosis.

1 Unadjusted Pearson correlation coefficients between food groups and dietary pattern score.

2 Standardized β regression coefficients for the associations between food groups and dietary pattern score.

3 Percentage of variation explained by each food group (column 1 value ×column 2 value×100).
